# Integrative bioinformatics and experimental analysis revealed down-regulated CDC42EP3 as a novel prognostic target for ovarian cancer and its roles in immune infiltration

**DOI:** 10.7717/peerj.12171

**Published:** 2021-09-15

**Authors:** Yuanliang Yan, Qiuju Liang, Zhijie Xu, Qiaoli Yi

**Affiliations:** 1 Department of Pharmacy, Xiangya Hospital, Central South University, Changsha, Hunan, China; 2 Department of Pathology, Xiangya Hospital, Central South University, Changsha, Hunan, China; 3 National Clinical Research Center for Geriatric Disorders, Xiangya Hospital, Central South University, Changsha, Hunan, China

**Keywords:** CDC42EP3, Immune infiltration, Ovarian cancer, N6-methyladenosine, Prognosis

## Abstract

Ovarian cancer is a significant clinical challenge as no effective treatments are available to enhance patient survival. Recently, N6-methyladenosine (m^6^A) RNA modification has been demonstrated to play a pivotal role in tumorigenesis and progression. However, the roles of m^6^A target genes in ovarian cancer haven’t been clearly illustrated. In this study, we presented a comprehensive bioinformatics and in vitro analysis to evaluate the roles of m^6^A target genes. Cell division cycle 42 effector protein 3 (CDC42EP3), one probable m6A target gene, was identified to be down-regulated in ovarian cancer tissues and cells. Meanwhile, quantitative PCR (qPCR) and western blot were used to confirm the down-regulated CDC42EP3 in ovarian cancer cells A2780 and TOV112D. The biological function of CDC42EP3 in ovarian cancer was further validated with several algorithms, such as PrognoScan, K-M plotter, LinkedOmics and TISIDB. These findings indicated that lower expression of CDC42EP3 was correlated with poor prognosis in patients with ovarian cancer. In addition, CDC42EP3 expression was significantly associated with a diverse range of tumor-infiltrating immune cells, including natural killer cells (NK), T central memory cells (Tcm), T gamma delta cells (Tgd), etc. Taken together, this study uncovered the potential roles of m^6^A target gene CDC42EP3 in the regulation of immune microenvironment in the ovarian cancer, and identified CDC42EP3 as a novel prognostic target.

## Introduction

Ovarian cancer is the eighth most frequently diagnosed cancer and the eighth leading cause of cancer death in women, with an estimated 314,000 new cases (3.4% of all cancer cases) and 207,000 deaths (4.7% of all cancer deaths) worldwide in 2020 (Sung et al., 2021). Despite the advances made in cancer screening, diagnosis and treatment methods during the past few decades, the five-year survival rate for ovarian cancer has changed modestly (Torre et al., 2018). Thus, discovering potential diagnostic markers and specific therapeutic targets are of importance to improve survival rate of ovarian cancer patients.

The tumor-infiltrating immune cells in the tumor microenvironment (TME) play pivotal roles in the initiation, progression, invasion, and metastasis of the malignant tumors. Poly (ADP-ribose) polymerase inhibitors (PARPi) have shown promising clinical benefits in ovarian cancer through influencing the TME and immune system response (Turinetto et al., 2021). The cell subtypes characterized by low expression of checkpoints and major histocompatibility complex (MHC) have been proved to be associated with the poor prognosis of ovarian cancer patients (Cong et al., 2020). However, comprehensive research on the tumor immune microenvironment of ovarian cancer is still rare and needs further exploration.

Recent studies have suggested the biofunctional roles of cell division cycle 42 effector protein 3 (CDC42EP3) in regulating actin cytoskeleton re-organization during cell shape changes, including pseudopodia formation (Farrugia & Calvo, 2016). In addition, CDC42EP3 also plays an important role in the occurrence and development of human cancers. Feng’s group revealed that CDC42EP3 was significantly up-regulated in colorectal cancer, and its high expression could accelerate cancer progression through regulating cell proliferation, apoptosis, and migration (Feng et al., 2021). In contrast, the expression of CDC42EP3 was down-regulated in peripheral blood monocyte from patients with chronic lymphocytic leukemia, which may be associated with impairment of phagocytosis (Maffei et al., 2013). However, the detailed function and mechanism of CDC42EP3 in the tumorigenesis and immune infiltration of ovarian cancer have not yet been investigated.

The purpose of this study was to investigate the potential roles of CDC42EP3 in ovarian cancer. The expression of CDC42EP3 was found to be down-regulated in ovarian cancer tissues and cells, and its low level was associated with poor prognosis. The results revealed that CDC42EP3 expression had significant prognostic value and could be a promising target for immune regulation in ovarian cancer.

## Material and Methods

### Cell culture

The human ovarian epithelial cell IOSE80 and ovarian cancer cells A2780 and TOV112D were obtained from Center for Molecular Medicine, Xiangya Hospital, Central South University. All cell lines were cultured in Dulbecco’s Modified Eagle’s Medium (DMEM, Gibco, USA) with 10% fetal bovine serum (FBS, Gibco, USA) and 1% penicillin/streptomycin (Gibco, USA). The cultures were placed in a sterile incubator maintained at 37 °C with 5% CO2.

### Western blot

To begin this procedure, cell lysates were processed with RIPA buffer supplemented with protease inhibitor. The total protein concentration was detected by BCA protein assay kit. Equivalent amounts of protein were loaded in each lane of SDS-polyacrylamide gel electrophoresis. Besides, the bands were transferred to PVDF membranes. Next, the membranes were blocked for 1 h at room temperature with TBST supplemented with 5% non-fat dry milk and then incubated at 4 °C overnight with primary antibodies. The primary antibodies displayed as follows: CDC42EP1 (1:500; Abcam, USA) and *β*-actin (1:5000; Santa Cruz, USA). Finally, protein bands were detected through the Immobilon Western Chemiluminescent HRP reagents (WBKLS0500; Millipore, USA).

### Quantitative PCR (qPCR)

Total RNA was extracted from disparate cell lines using TRIzol reagent (Invitrogen, USA) following the manufacturer’s protocol and then converted to cDNA using a PrimeScriptTM RT reagent kit (6210; Takara, China). The qPCR assay was performed with iTaqTM Universal SYBR green Supermix (1725121, Bio-Rad, USA) to determine the relative RNA levels. *β*-actin was used as an internal control for quantification of each gene. The sequences of gene primers displayed as follows: CDC42EP3 forward: 5′-AGCAGTCTGTTGGAGAATGGG-3′and reverse: 5′-AGGAGGGAACCTGTAAGGTCAG-3′; *β*-actin forward: 5′-CATGTACGTTGCTATCCAGGC-3′and reverse, 5′-CTCCTTAATGTC ACGCACGAT-3′. The relative expression levels of RNAs were determined using the 2-ΔΔCT method (Buford et al., 2020; Kiss et al., 2019; Pandelides et al., 2020).

### Data acquisition and processing

The integrative bioinformatics analysis of differential expressed genes was obtained from several bioinformatics databases (Table 1).

The gene expression profiles from two public datasets regarding ovarian cancer were obtained from the Oncomine datasets (Rhodes et al., 2007), Yoshihara Ovarian and Bonome Ovarian. The GEO2R, an R-based web application (Barrett et al., 2013), was applied to assess the co-differential expressed genes in these two datasets. The screening criteria to identify the differential expression genes were as follows: *p*-value < 0.01 and — log_2_Fold Change —≥ 1.5. In addition, the N^6^-methyladenosine (m^6^A) target genes of ovarian cancer were acquired from the RNA modification-associated variants database (RMVar) (Luo et al., 2021).

UALCAN (Chandrashekar et al., 2017) integrates the RNA-seq data and clinical information of pan-cancer types from The Cancer Genome Atlas (TCGA). This database could enable researchers to explore the expression level of candidate genes between primary tumor and normal tissue samples.

PrognoScan (Mizuno et al., 2009) is utilized to analyze the relation between gene expression and clinical prognosis of patients, including overall survival (OS). In this research, PrognoScan was applied for the survival analysis of differentially expressed genes (DEGs) in patients with ovarian cancer. In addition, the Kaplan Meier (K-M) plotter (Nagy, Munkacsy & Gyorffy, 2021) is capable to assess the effect of candidate genes on the prognosis in pan-cancer. The K-M survival curves were applied to exhibit the prognostic effects of CDC42EP3 in ovarian cancer patients.

**Table 1:** The integrative bioinformatics analyzed in this study.

Database	URL	Refs.
Oncomine	https://www.oncomine.org/resource/main.html	Rhodes et al. (2007)
RMVar	http://www.rmvar.renlab.org/browse.html	Luo et al. (2021)
UALCAN	http://ualcan.path.uab.edu/	Chandrashekar et al. (2017)
PrognoScan	http://dna00.bio.kyutech.ac.jp/PrognoScan/index.html	Mizuno et al. (2009)
Kaplan-Meier Plotter	https://kmplot.com/analysis/	Nagy, Munkacsy & Gyorffy (2021)
LinkedOmics	http://linkedomics.org/login.php	Vasaikar et al. (2018)
TISIDB	http://cis.hku.hk/TISIDB	Ru et al. (2019)
TNMplot	https://tnmplot.com/	Bartha & Gyorffy (2021)

LinkedOmics (Vasaikar et al., 2018) provides a unique platform to analyze multi-omics data across pan-cancer types. The DEGs associated with CDC42EP3 were screened from the TCGA-OV cohort through LinkFinder module in this database. The statistical method used in correlation analysis was Pearson correlation and the results of correlation were presented in volcano plot and heat maps, respectively. The LinkInterpreter module was performed to analyze the Gene Ontology molecular function (GO_MF) and Kyoto Encyclopedia of Genes and Genomes (KEGG) pathway.

The TISIDB (Ru et al., 2019) is a web portal for tumor and immune system interplay, which contains multiple heterogeneous data. Here, TISIDB was employed to investigate the roles of CDC42EP3 in several immune-associated signaling pathways, such as tumor-infiltrating immune cells, immunomodulators, chemokines and chemokine receptors.

### Statistical analyses

All experimental findings were shown as mean ± standard deviation (SD). Student’s *t*-test was used to explore the difference between two groups. *P* < 0.05 was considered as statistically significant difference.

## Results

### Identification of differentially expressed genes

To screen for DEGs in ovarian cancer, we compared normal ovary tissues and tumor samples in two datasets from Oncomine, Yoshihara Ovarian and Bonome Ovarian. According to the filtering conditions of *p*-value < 0.01 and —log_2_Fold Change—≥1.5, we identified 225 up-regulated genes in Bonome Ovarian and 1085 up-regulated genes in Yoshihara Ovarian, respectively. Meanwhile, 536 genes in Bonome Ovarian and 623 genes in Yoshihara Ovarian have been identified to be significantly down-regulated in ovarian cancer tissues (Table S1), and 757 m^6^A target genes of ovarian cancer acquired from the RMVar database were shown in Table S2.

**Figure 1:**
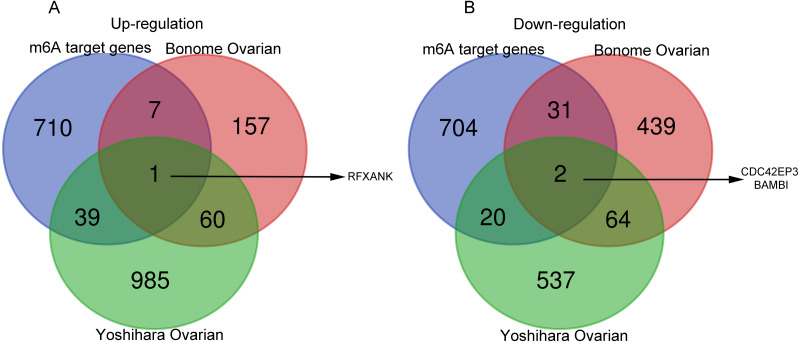
Venn diagrams of gene expression profiles. (A) RFXANK was identified as the only one up-regulated overlapping gene among Yoshihara Ovarian, Bonome Ovarian and m^6^A target gene set. (B) CDC42EP3 and BAMBI were recognized as the down-regulated overlapping genes among Yoshihara Ovarian, Bonome Ovarian and m^6^A target gene set. The number in each overlapping area represents the number of co-DEGs.

Next, the co-DEGs between Yoshihara Ovarian and Yoshihara Ovarian were analyzed by Venn diagram (http://bioinformatics.psb.ugent.be/webtools/Venn/). Studies have demonstrated that the m^6^A RNA modification, a reversible and dynamic epigenetic modification, affects the RNA metabolism and homeostasis, and plays critical functions in multiple of important physiological and pathological bioprocesses, including tumorigenesis and progression (Xu et al., 2020a). In order to investigate the possible roles of m^6^A target genes in ovarian cancer, Venn diagram was used to display the overlapping genes between co-DEGs and m^6^A target genes. The results showed that one m^6^A target gene, regulatory factor X subunit B (RFXANK), was highly-expressed, and two m^6^A target genes, CDC42EP3 and BMP and activin membrane bound inhibitor (BAMBI), were lowly-expressed (Figs. 1A–1B). These three genes were presumed to have a significance impact on the development of ovarian cancer.

### CDC42EP3 shows the promising prognostic value in ovarian cancer

**Figure 2:**
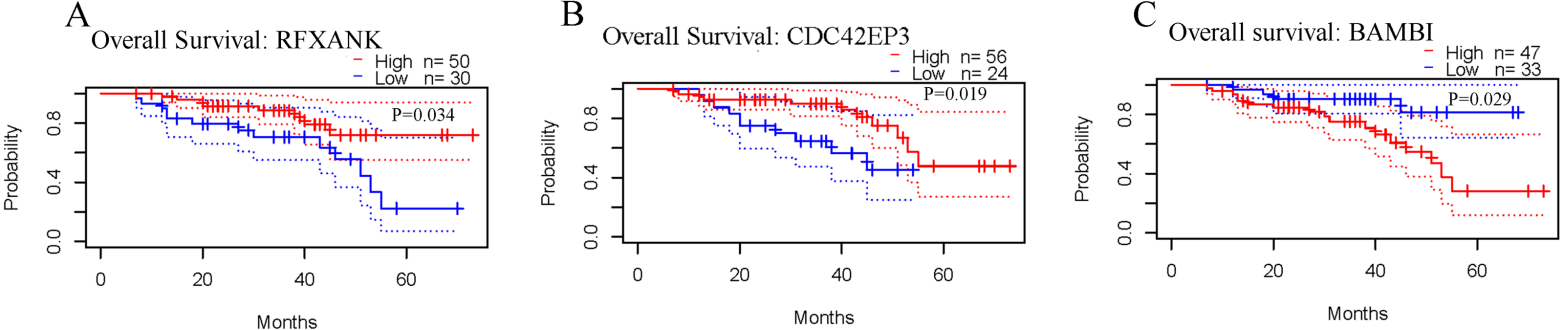
Prognostic values of RFXANK, CDC42EP3 and BAMBI in ovarian cancer. (A–C) PrognoScan database was used to analyze the kaplan-Meier curves of overall survival for patients with high or low expression levels of RFXANK, CDC42EP3 and BAMBI.

The correlations between the expression levels of RFXANK, CDC42EP3 and BAMBI and prognosis in ovarian cancer patients were analyzed by the PrognoScan database. The results revealed that patients with high level of RFXANK and CDC42EP3 displayed favorable OS (Figs. 2A–2B). However, patients with high level of BAMBI displayed poor OS (Fig. 2C). Given the up-regulated RFXANK and down-regulated CDC42EP3 and BAMBI in two independent ovarian cancer datasets from Oncomine (Figs. 1A–1B), we expected CDC42EP3 as a potential prognostic target that needs further investigation.

### Associations between CDC42EP3 expression and clinicopathological parameters in ovarian cancer patients

**Figure 3:**
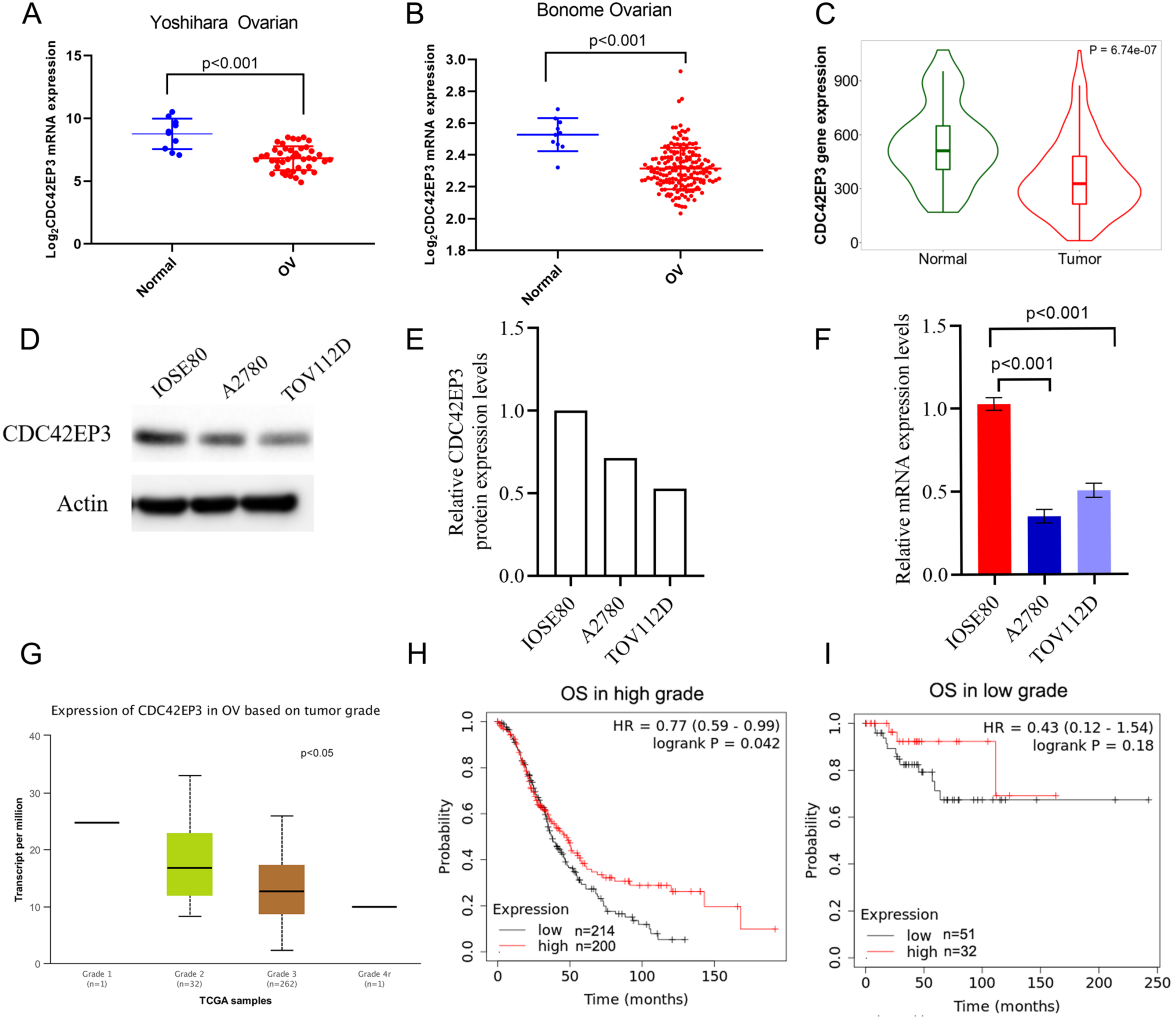
Associations between down-regulated CDC42EP3 and clinicopathological parameters in ovarian cancer patients. (A–C) Compared to normal ovary tissues, CDC42EP3 was significantly down-regulated in ovarian cancer tissues. (D–F) qPCR and western blot were used to confirm the down-regulation of CDC42EP3 expression in ovarian cancer cells A2780 and TOV112D compared with the normal ovarian epithelial cell IOSE80. (G) Association of CDC42EP3 level with tumor grade. (H–I) Kaplan–Meier curves was conducted to explore the roles of CDC42EP3 expression on the OS in low-grade and high-grade ovarian cancer patients.

The expression profiles of CDC42EP3 were further confirmed using several independent bioinformatics databases, such as TNM plot (Bartha & Gyorffy, 2021) and UALCAN. Firstly, two above-mentioned datasets, Yoshihara Ovarian and Bonome Ovarian, indicated that CDC42EP3 transcriptional level in ovarian cancer samples was significantly lower than adjacent noncancerous tissues (Figs. 3A–3B). Next, TNM plot revealed that CDC42EP3 mRNA expression levels were significantly down-regulated in ovarian cancer samples (Fig. 3C). In addition, qPCR and western blot were used to confirm the down-regulation of CDC42EP3 expression in ovarian cancer cells A2780 and TOV112D compared with the normal ovarian epithelial cell IOSE80 (Figs. 3D–3F). All these data revealed that the expression of CDC42EP3 was significantly downregulated in ovarian cancer tissues and cell lines.

Furthermore, we analyzed the correlation between CDC42EP3 expression levels and clinicopathological features of ovarian cancer patients. According to UCLCAN database, we discovered that the higher tumor grade, the lower expression levels of CDC42EP3 in ovarian cancer (Fig. 3G). However, the Xiantao tool (http://www.xiantao.love/products) was analyzed to reveal that CDC42EP3 expression has no correlation with the other clinical parameters, including tumor status, stage, age and invasion (Table 2). In addition, the K-M plotter uncovered that the expression of CDC42EP3 was significantly positively correlated with OS in high-grade tumor patients (*p* < 0.05) (Fig. 3H). However, the correlation between CDC42EP3 level and OS in low-grade patients was of no statistical significance (Fig. 3I). Collectively, these results indicate that CDC42EP3 could be a potential prognostic biomarker for ovarian cancer, especially like the high-grade patients.

**Table 2:** Demographic characteristics of CDC42EP3 expression in ovarian cancer.

Characteristics	Total(N)	Odds ratio	*P* value
Stage (Stage III&Stage IV vs. Stage I&Stage II)	376	1.432 (0.624–3.404)	0.401
Primary therapy outcome (CR&PR vs. PD&SD)	308	1.139 (0.618–2.112)	0.676
Age (> 60 vs. ≤ 60)	379	0.817 (0.545–1.225)	0.329
Venous invasion (No vs. Yes)	105	0.754 (0.338–1.658)	0.484
Lymphatic invasion (No vs. Yes)	149	1.674 (0.839–3.385)	0.146
Tumor residual (NRD vs. RD)	335	1.391 (0.813–2.400)	0.230
Tumor status (Tumor free vs. With tumor)	337	1.241 (0.736–2.104)	0.420

### CDC42EP3 co-expression network in ovarian cancer

**Figure 4:**
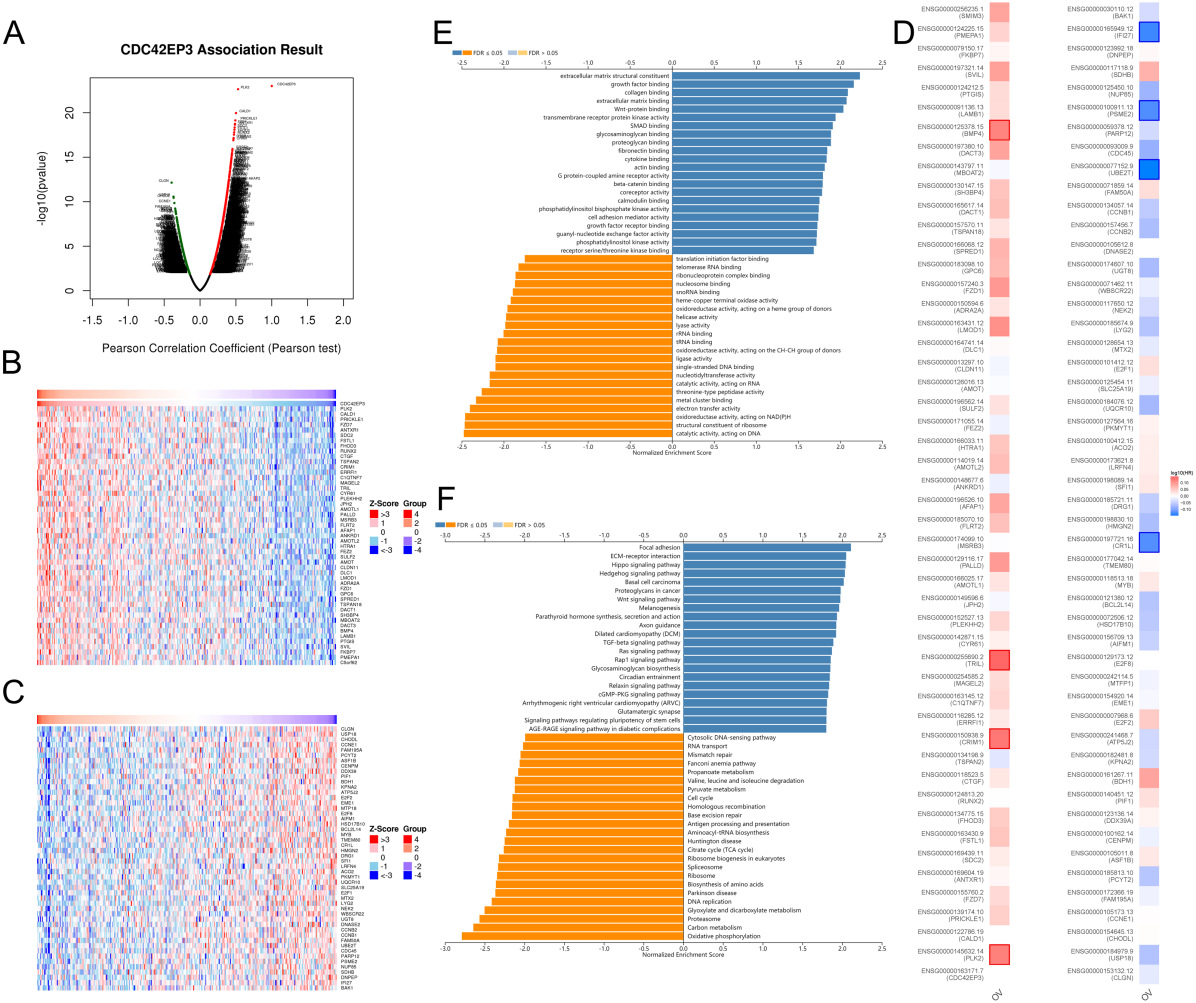
The co-expression genes with CDC42EP3 in ovarian cancer by the LinkedOmics database. (A) The significantly associated genes with CDC42EP3 analyzed by Pearson correlation in ovarian cancer cohort. (B–C) Heatmaps of the top 50 genes positively and negatively correlated with CDC42EP3 in ovarian cancer, respectively. Red dots indicate the positively correlated genes and blue dots indicate the negatively correlated genes. (D) Survival maps depicting the top 50 genes positively and negatively related to CDC42EP3 in ovarian cancer, respectively. (E–F) GO annotations and KEGG pathways of CDC42EP3-associated network in ovarian cancer cohort.

In order to explore the biological functions of CDC42EP3 in ovarian cancer, we performed the co-expression pattern of CDC42EP3 screened from the TCGA-OV cohort by the LinkFinder module of LinkedOmics. As shown in Fig. 4A, 4227 genes (red dots) were positively correlated with CDC42EP3, and 3122 genes (green dots) were negatively correlated with CDC42EP3. Figures 4B–4C showed the heatmaps of top 50 genes positively and negatively associated with CDC42EP3, respectively (Tables S3–S4). 14/50 positively correlated genes had protective hazard ratio (HR), while 13/50 genes negatively correlated with CDC42EP3 had unfavorable HR (Fig. 4D).

To further elaborate the potential biological roles of CDC42EP3 in ovarian cancer development, we performed GO and KEGG functional enrichment analysis. GO enrichment analysis revealed that co-expression genes of CDC42EP3 mainly participated in extracellular matrix structural constituent, growth factor binding, collagen binding, extracellular matrix binding, Wnt-protein binding, etc (Fig. 4E). KEGG pathway analysis indicated these genes were significantly related to several cancer-associated signaling pathways, such as focal adhesion, extracellular matrix (ECM)-receptor interaction, Hippo signaling pathway, hedgehog signaling pathway, basal cell carcinoma, Wnt signaling pathway, etc (Fig. 4F). These findings suggested that important functions of CDC42EP3-associated network in ovarian cancer.

### Relation between CDC42EP3 with immune molecules

**Figure 5:**
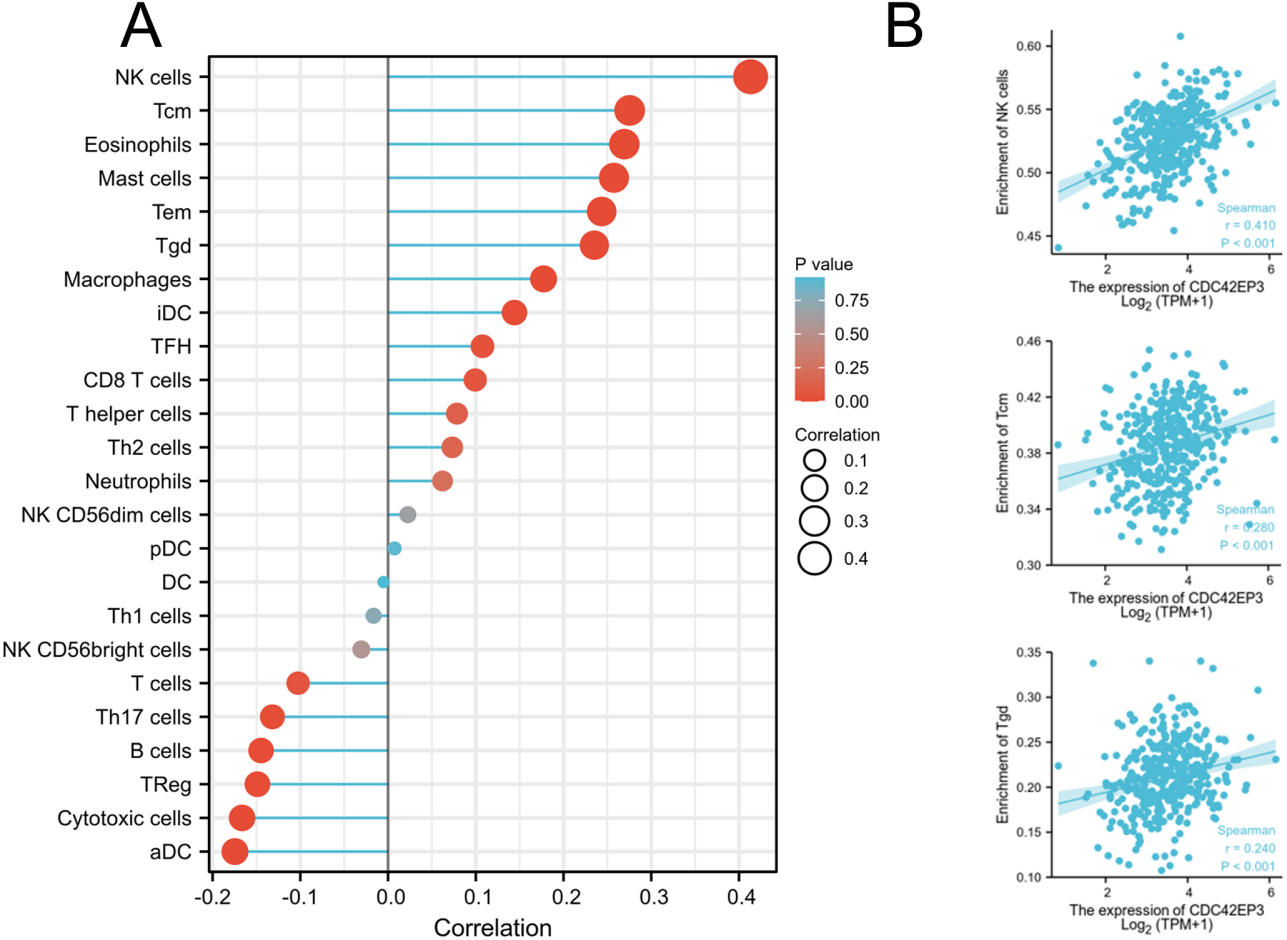
Correlation of CDC42EP3 expression with tumor-infiltrating immune cells in ovarian cancer. (A) Lollipop of correlation between CDC42EP3 expression and immune cells by the Xiantao Tool. (B) The cross-validated correlation between CDC42EP3 expression and several immune cells, such as NK, Tcm and Tgd cells.

We explored whether CDC42EP3 expression can affect the abundance of tumor-infiltrating lymphocytes (TILs) in ovarian cancer using Xiantao Tool. The significantly positive relations could be obtained between CDC42EP3 expression and several tumor-infiltrating immune cells, including natural killer cells (NK), T central memory cells (Tcm) and T gamma delta cells (Tgd) (Figs. 5A–5B). Similar results were obtained by analyzing the immune-related signatures of 28 TIL types through TISIDB algorithm (Figs. S1A–S1B).

Furthermore, we used TISIDB database to analyze the correlation between CDC42EP3 expression and other immune signatures, including immunostimulators, immunoinhibitors, chemokines and chemokine receptors. Figs. S2A–S2B exhibited the positively correlated immunostimulators with CDC42EP3, including 5′-nucleotidase ecto (NT5E), C-X-C motif chemokine ligand 12 (CXCL12), tumor necrosis factor superfamily member 4 (TNFSF4), and TNF receptor superfamily member 8 (TNFRSF8). Figures S2C–S2D uncovered the negatively correlated immunoinhibitors with CDC42EP3, including galectin 9 (LGALS9), indoleamine 2,3-dioxygenase 1 (IDO1), cluster of differentiation 274 (CD274) and V-set domain containing T cell activation inhibitor 1 (VTCN1). Figures S3A–S3B showed the significant associations between CDC42EP3 expression and chemokines, such as C-C motif chemokine ligand 14 (CCL14). Figures S3C–S3D revealed the associations between CDC42EP3 expression and chemokine receptors, including C-C motif chemokine receptor 1 (CXCR1). Generally, we concluded that CDC42EP3 might participated in modulating diverse of immunoregulatory molecules in ovarian cancer, which could affect the infiltration and functions of immune cells in TME.

## Discussion

Our research aimed to explore the m^6^A target genes in the occurrence and development of ovarian cancer. Intriguingly, through the integrative bioinformatics and experimental analysis, the expression of CDC42EP3 was found to be down-regulated in ovarian cancer tissues and cells, and its low level was associated with poor prognosis.

The m^6^A modification in messenger RNAs is the most widespread epitranscriptome modification involving in the tumorigenesis and development (Huang et al., 2020a; Zhang et al., 2020). RNA m^6^A modification is regulated by three major epigenetic modifiers, including methyltransferases (writers), demethylases (erasers) and binding proteins (readers) (Geng et al., 2020). Hua’s group unveiled that methyltransferase-like 3 (METTL3) was up-regulated in ovarian cancer and promoted growth and invasion of ovarian cancer cells *via* stimulating the receptor tyrosine kinase AXL translation (Hua et al., 2018). In addition, Huang et al. disclosed that the fat mass- and obesity-associated protein (FTO), an m^6^A demethylase, was down-regulated in ovarian cancer stem cells (CSC) and inhibited the cell self-renewal by blocking cAMP signaling (Huang et al., 2020b). Xu and the colleague revealed that F-box and WD repeat domain-containing 7 (FBW7) suppressed ovarian cancer progression through targeting the m^6^A binding protein YTH domain family 2 (YTHDF2) (Xu et al., 2021). Nevertheless, more research should be carried out to clarify the detailed mechanisms of m^6^A modification in ovarian tumorigenesis and development.

Cell division cycle 42 (CDC42), a family member of the Rho GTPase proteins, modulates a variety of essential biological processes, including cell polarization, cytoskeleton remodeling, vesicular trafficking, and pseudopodia formation (Maldonado & Dharmawardhane, 2018). Like other Rho family members, CDC42 possesses two conformational states, GDP-bound inactive state and GTP-bound active state. Activated CDC42 exerts important physiological and pathological functions *via* interacting with downstream effectors, like CDC42/Rac interactive binding motif (CRIB) (Farrugia & Calvo, 2016). CDC42EP3, one of the CDC42 effector proteins, participates in cell actin cytoskeleton re-organization. The current studies concerning the biological roles of CDC42EP3 have been mainly focused on its regulatory effect on tumor progression. The expression level of CDC42EP3 was up-regulated in primary cultured hepatocellular carcinoma cells and can enhance the cellular migratory ability *in vitro* (Lin & Chuang, 2012). It has also been shown that the up-regulated CDC42EP3 in colorectal cancer tissues can promote tumor growth by regulating cell proliferation, apoptosis, and migration (Feng et al., 2021). Similarly, Chen et al. (2021) revealed that up-regulated CDC42EP3 in gastric cancer tissues facilitated tumor development and progression. Conversely, down-regulated CDC42EP3 was found in patients with chronic lymphocytic leukemia (Maffei et al., 2013). Likewise, Hsia et al. (2015) reported that CDC42EP3 was down-regulated in cantharidin-treated lung cancer cells. However, the detailed roles of CDC42EP3 in human gynecological tumors, especially ovarian cancer, have been rarely studied. In this research, we firstly indicated the clinical prognostic value and potentially functional roles of CDC42EP3 in ovarian cancer through a comprehensive bioinformatics analysis.

GO and KEGG functional enrichment analysis indicated the co-expression genes of CDC42EP3 mainly involved in several cancer-associated signaling pathways, such as focal adhesion, ECM-receptor interaction and Hippo signaling pathway. Focal adhesion connects cytoskeleton within ECM, participating in the regulation of cell adhesion, proliferation and apoptosis. Focal adhesion kinase (FAK) is a widely expressed non-receptor tyrosine kinase participating in integrin-mediated signal transduction. Its overexpression and activation are frequently identified in multiple cancers, including ovarian cancer, and are associated with poor clinical outcome (Levy et al., 2019). The invasive phenotype of ovarian cancer cells was found to be closely correlated to ECM-receptor interaction (Wang et al., 2020). The Hippo signaling pathway controls organ size by regulating cell proliferation and apoptosis (Yu et al., 2012). Increasingly evidence has confirmed that dysregulation of Hippo pathway is involved in cancer immunosuppressive microenvironment (Yang et al., 2020). Therefore, CDC42EP3 might participate in malignant biological phenotypes of ovarian cancer through the afore-mentioned signaling pathways. However, the specific biological mechanism still needs further experimental verification.

The TME is defined as an intricate and multicellular environment that is clearly associated with cancer characteristic and therapy response (Jia, Liu & Shan, 2020; Wei et al., 2020). The TME typically comprises extracellular matrix, stromal cells, immune cells and blood and lymphatic vascular networks (Horowitz et al., 2020). Recently, accumulating evidence has demonstrated that TME plays a crucial role in tumor progression and therapeutic resistance by facilitating immunosuppression and limiting anticancer immune responses. Immunotherapies, including checkpoint blockade therapy, oncolytic virus, and adoptive cell therapy, are shifting the tumor treatment landscape and outlook (Da Silva et al., 2019; Xu et al., 2020b). Advanced ovarian cancer is the most lethal gynecological tumor with a particularly poor prognosis, which makes it urgent to explore novel therapeutic strategies. In this research, the correlation between CDC42EP3 and immune signatures was cross-validated by the TISIDB database and Xiantao Tool. It was found that CDC42EP3 had the most relevance with several TILs, including NK cell, Tcm_CD4 and Tgd. NK-based immunotherapy strategy, especially cytokine-induced killer (CIK) cells, has been extensively studied for decades (Sarivalasis et al., 2021). The clinical efficacy of CIK cells was evaluated in patients with advanced epithelial ovarian cancer in a phase 3 clinical trial. The results showed an improved progression free survival (PFS) in the treatment (Liu et al., 2014). Furthermore, CDC42EP3 had the highest correlation with immunostimulators, including NT5E, TNFSF4, CXCL12 and TNFRSF8, and immunoinhibitors, including LGALS9, IDO1, CD274 and VTCN1. NT5E, also designated CD73, is an ecto-nucleotidase that catalyzes extracellular ATP to adenosine and plays a critical role in the maintenance of immune homeostasis. High CD73 expression was significantly correlated with shorter OS in ovarian cancer (Jiang et al., 2018). Programmed death-ligand 1 (PD-L1), also known as CD274, is high expressed in ovarian cancer. However, the single agent targeting PD-1/PD-L1 has shown modest efficiency in treating ovarian cancer (Pujade-Lauraine, 2017). Currently, immunotherapy combined with other anticancer therapies, such as chemotherapy, radiotherapy and targeted therapy, are underway. Together, these findings suggest that CDC42EP3, which is correlated with immune-associated molecules, can serve as a potential immunotherapeutic target for treatment of ovarian cancer.

Nonetheless, several limitations in this study need to be acknowledged. First, the data collected from several public database were retrospective. Thus, the results need to be further validated in large-scale prospective studies. Moreover, more functional experiments as well as clinical trials are warranted to explore the clinical value of CDC42EP3 in patients with ovarian cancer.

## Conclusion

In conclusion, this is the first study to investigate the clinical prognostic roles of CDC42EP3 in ovarian cancer. CDC42EP3 is down-regulated in ovarian cancer tissues and cells, and may serve as a promising prognostic biomarker for ovarian cancer patients. Furthermore, the expression of CDC42EP3 is obviously associated with TILs, which profoundly affect the prognosis of ovarian cancer. Therefore, our findings indicated that CDC42EP3 might play key roles in TME, and provide a novel target for immunotherapy.

##  Supplemental Information

10.7717/peerj.12171/supp-1 Supplemental Information 1 Correlation of CDC42EP3 expression with tumor-infiltrating immune cells in patients with ovarian cancer (A) Heatmap indicated the correlation between CDC42EP3 expression and immune cells analyzed by TISIDB. (B) The cross-validated correlation between CDC42EP3 expression and several immune cells, such as NK, Tcm and Tgd cells.

10.7717/peerj.12171/supp-2 Supplemental Information 2 Correlation of CDC42EP3 expression with immunomodulators in ovarian cancer (A) The correlation between CDC42EP3 expression and multiple of immunostimulators. (B) The top four immunostimulators highly correlated with CDC42EP3 expression. (C) The correlation between CDC42EP3 expression and multiple of immunoinhibitors. (D) The top four immunoinhibitors highly correlated with CDC42EP3 expression.

10.7717/peerj.12171/supp-3 Supplemental Information 3 Correlation of CDC42EP3 expression with chemokines and chemokine receptors in ovarian cancer (A) The correlation between CDC42EP3 expression and chemokines. (B) The significant associations between CDC42EP3 expression and CCL14. (C) The correlation between CDC42EP3 expression and chemokine receptors. (D) The significant associations between CDC42EP3 expression and CXCR1.

10.7717/peerj.12171/supp-4 Supplemental Information 4 Differentially expressed genes

10.7717/peerj.12171/supp-5 Supplemental Information 5 The m6A target genes of ovarian cancer acquired from the RMVar database

10.7717/peerj.12171/supp-6 Supplemental Information 6 The top 50 genes positively correlated with CDC42EP3 in LinkedOmics

10.7717/peerj.12171/supp-7 Supplemental Information 7 The top 50 genes negatively correlated with CDC42EP3 in LinkedOmics

10.7717/peerj.12171/supp-8 Supplemental Information 8 Original image of western blot Western blot confirmed the down-regulation of CDC42EP3 expression in ovarian cancer cells A2780 and TOV112D.

10.7717/peerj.12171/supp-9 Supplemental Information 9 Raw data of qPCR qPCR confirmed the down-regulation of CDC42EP3 expression in ovarian cancer cells A2780 and TOV112D.

## Additional Information and Declarations

### Data Availability

The following information was supplied regarding data availability: The raw measurements are available in the Supplementary Files.
