# Supplementary figures and images for: Integrative bioinformatics and experimental analysis revealed down-regulated CDC42EP3 as a novel prognostic target for ovarian cancer and its roles in immune infiltration
Source: PeerJ. 2021 Sep 15;9:e12171. doi: 10.7717/peerj.12171 (PMC8449529; doi:10.7717/peerj.12171)

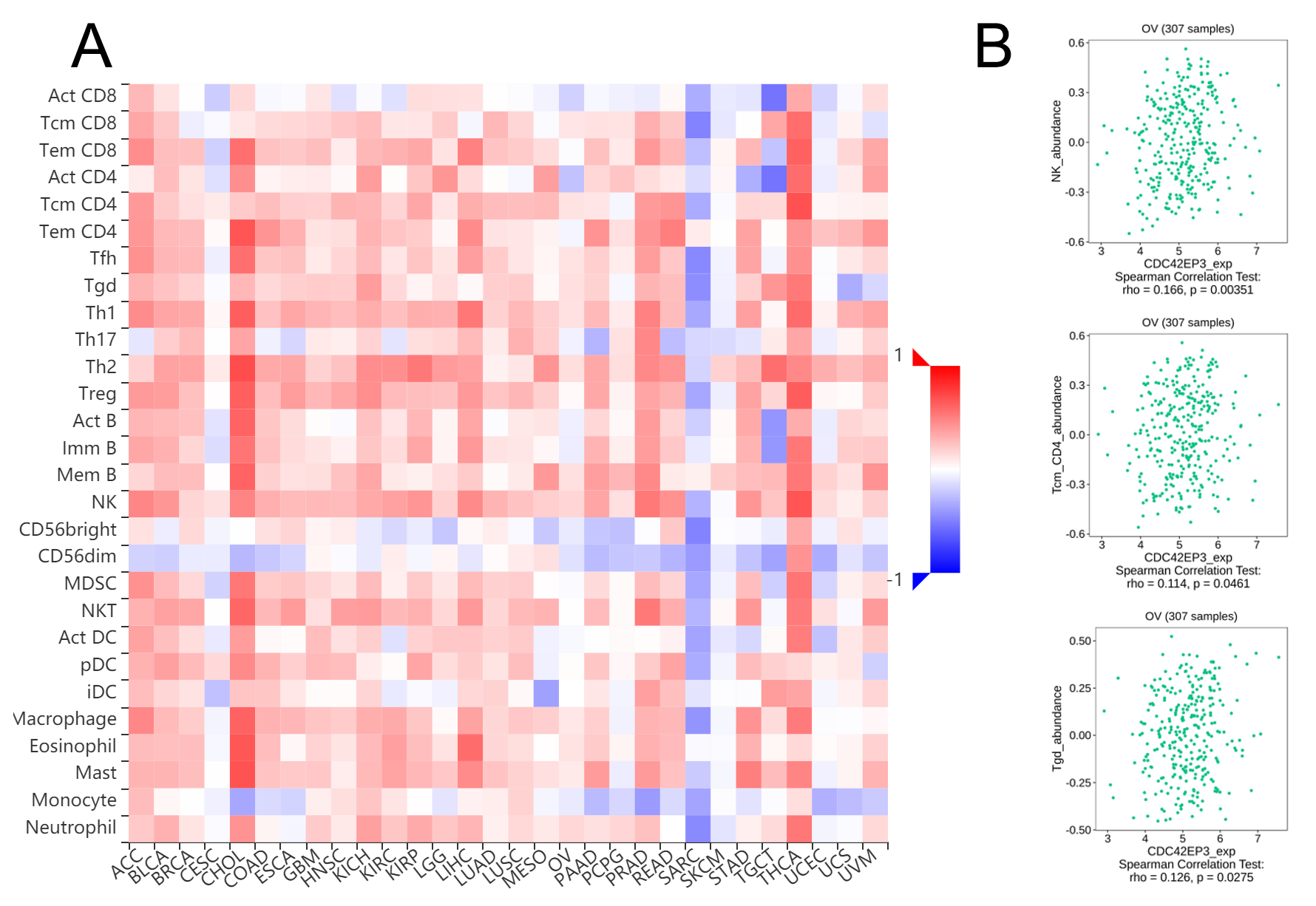

Supplement: Supplemental Information 1 — (A) Heatmap indicated the correlation between CDC42EP3 expression and immune cells analyzed by TISIDB. (B) The cross-validated correlation between CDC42EP3 expression and several immune cells, such as NK, Tcm and Tgd cells. [file peerj-09-12171-s001.png]

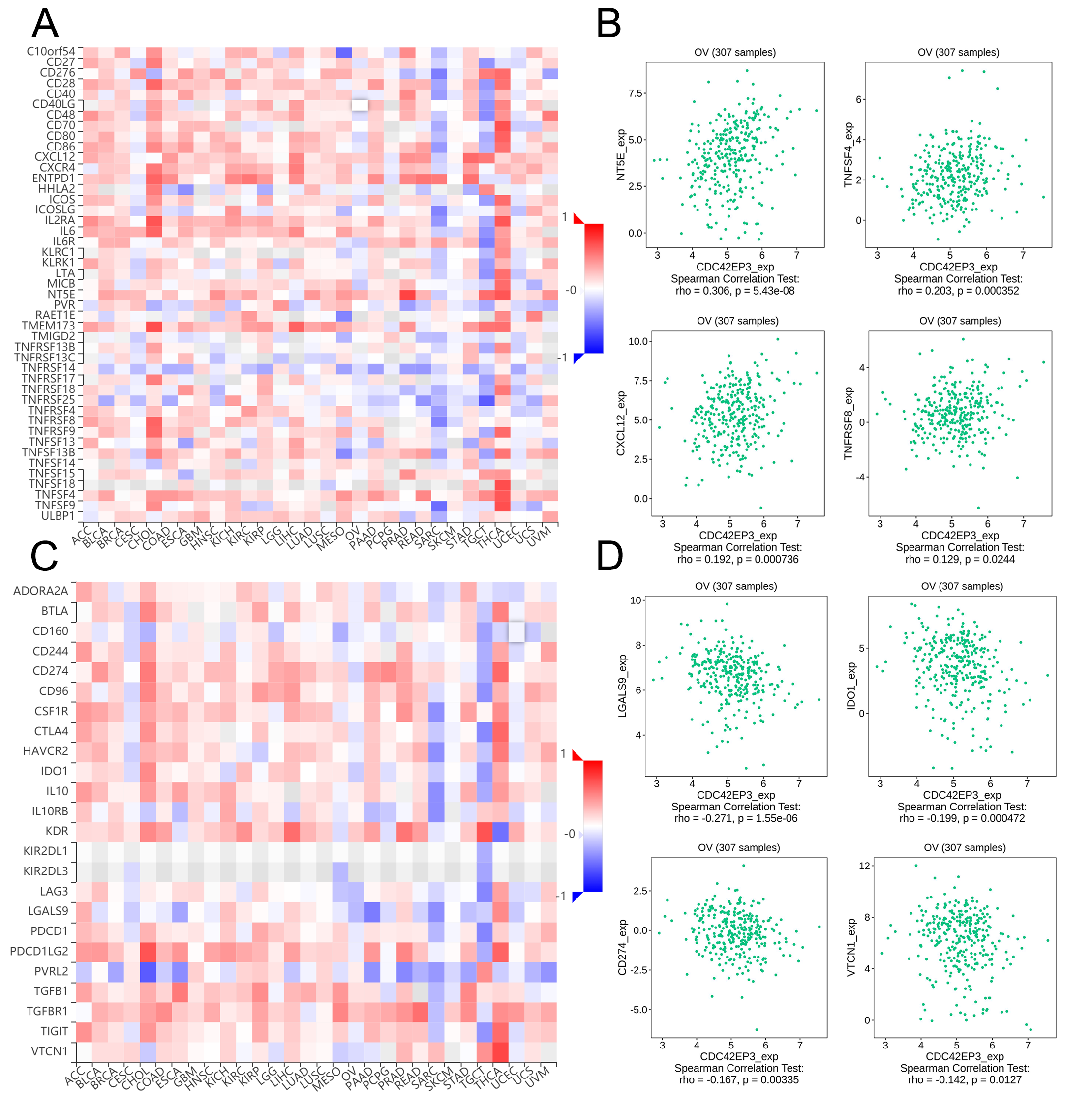

Supplement: Supplemental Information 2 — (A) The correlation between CDC42EP3 expression and multiple of immunostimulators. (B) The top four immunostimulators highly correlated with CDC42EP3 expression. (C) The correlation between CDC42EP3 expression and multiple of immunoinhibitors. (D) The top four immunoinhibitors highly correlated with CDC42EP3 expression. [file peerj-09-12171-s002.png]

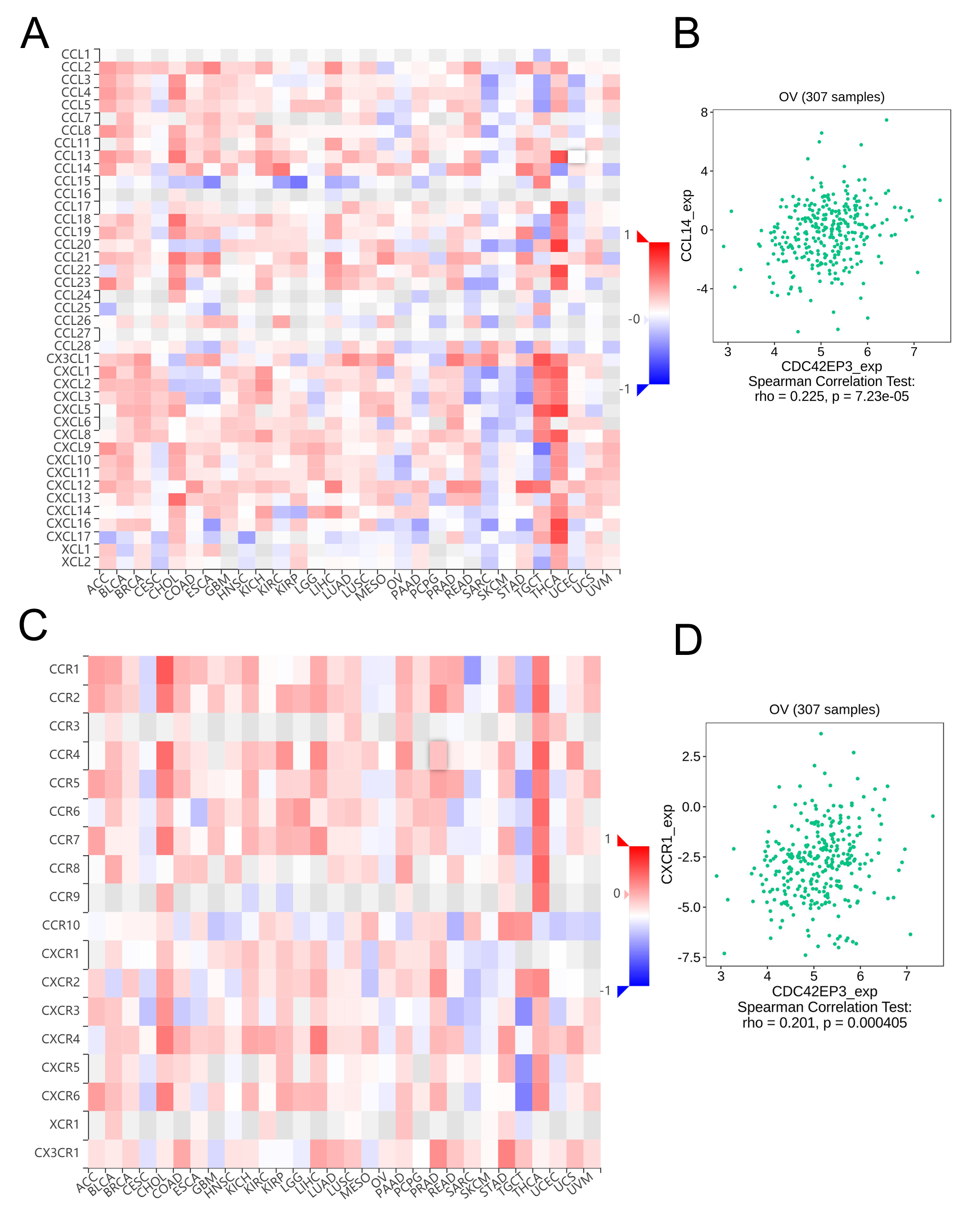

Supplement: Supplemental Information 3 — (A) The correlation between CDC42EP3 expression and chemokines. (B) The significant associations between CDC42EP3 expression and CCL14. (C) The correlation between CDC42EP3 expression and chemokine receptors. (D) The significant associations between CDC42EP3 expression and CXCR1. [file peerj-09-12171-s003.png]

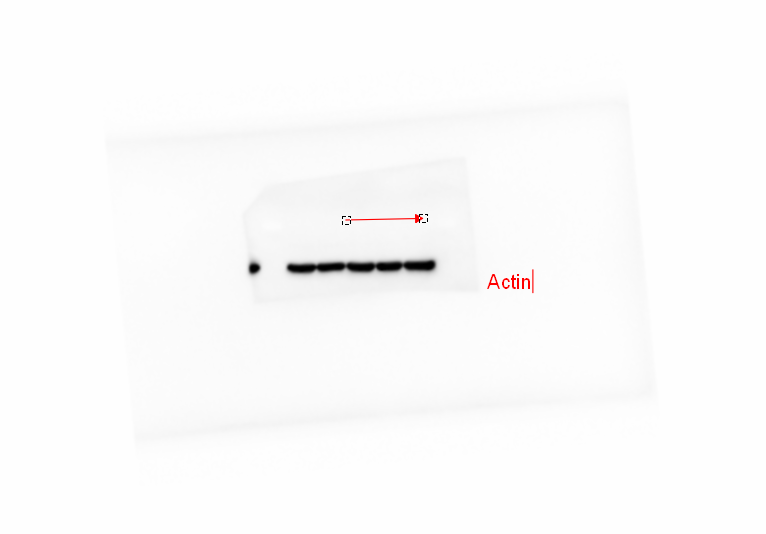

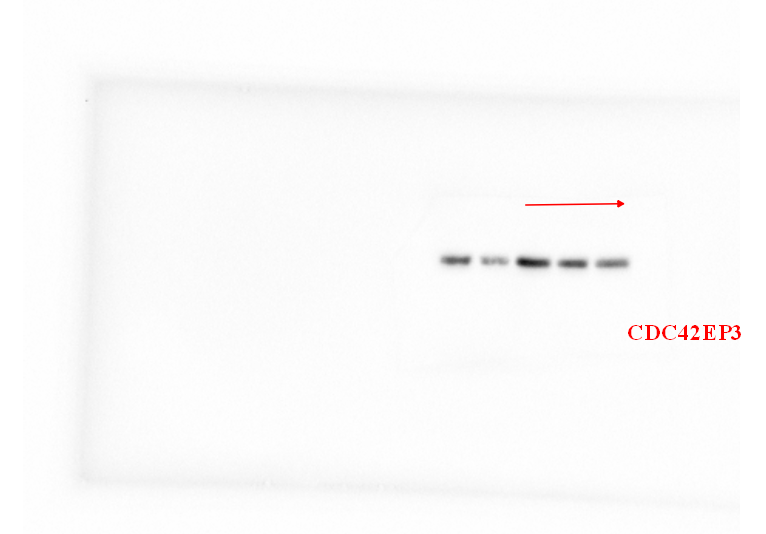

Supplement: Supplemental Information 8 — Western blot confirmed the down-regulation of CDC42EP3 expression in ovarian cancer cells A2780 and TOV112D. [file peerj-09-12171-s008.docx]
