# Supplementary material for: Integrative bioinformatics and experimental analysis revealed down-regulated CDC42EP3 as a novel prognostic target for ovarian cancer and its roles in immune infiltration
Source: PeerJ. 2021 Sep 15;9:e12171. doi: 10.7717/peerj.12171 (PMC8449529; doi:10.7717/peerj.12171)
Supplement: Supplemental Information 6 [file peerj-09-12171-s006.docx]

**Supplementary Table S3. The top 50 genes positively correlated with CDC42EP3 in LinkedOmics**

| CDC42EP3 | PLK2 | CALD1 | PRICKLE1 | FZD7 | ANTXR1 | SDC2 | FSTL1 | FHOD3 | RUNX2 |
| --- | --- | --- | --- | --- | --- | --- | --- | --- | --- |
| CTGF | TSPAN2 | CRIM1 | ERRFI1 | C1QTNF7 | MAGEL2 | TRIL | CYR61 | PLEKHH2 | JPH2 |
| AMOTL1 | PALLD | MSRB3 | FLRT2 | AFAP1 | ANKRD1 | AMOTL2 | HTRA1 | FEZ2 | SULF2 |
| AMOT | CLDN11 | DLC1 | LMOD1 | ADRA2A | FZD1 | GPC6 | SPRED1 | TSPAN18 | DACT1 |
| SH3BP4 | MBOAT2 | DACT3 | BMP4 | LAMB1 | PTGIS | SVIL | FKBP7 | PMEPA1 | C5orf62 |
