# Supplementary material for: Integrative bioinformatics and experimental analysis revealed down-regulated CDC42EP3 as a novel prognostic target for ovarian cancer and its roles in immune infiltration
Source: PeerJ. 2021 Sep 15;9:e12171. doi: 10.7717/peerj.12171 (PMC8449529; doi:10.7717/peerj.12171)
Supplement: Supplemental Information 7 [file peerj-09-12171-s007.docx]

**Supplementary Table S4. The top 50 genes negatively correlated with CDC42EP3 in LinkedOmics**

| CLGN | USP18 | CHODL | CCNE1 | FAM195A | PCYT2 | ASF1B | CENPM | DDX39 | PIF1 |
| --- | --- | --- | --- | --- | --- | --- | --- | --- | --- |
| BDH1 | KPNA2 | ATP5J2 | E2F2 | EME1 | MTP18 | E2F8 | AIFM1 | HSD17B10 | BCL2L14 |
| MYB | TMEM80 | CR1L | HMGN2 | DRG1 | SFI1 | LRFN4 | ACO2 | PKMYT1 | UQCR10 |
| SLC25A19 | E2F1 | MTX2 | LYG2 | NEK2 | WBSCR22 | UGT8 | DNASE2 | CCNB2 | CCNB1 |
| FAM50A | UBE2T | CDC45 | PARP12 | PSME2 | NUP85 | SDHB | DNPEP | IFI27 | BAK1 |
